# Echo-Level SAR Imaging Simulation of Wakes Excited by a Submerged Body

**DOI:** 10.3390/s24041094

**Published:** 2024-02-07

**Authors:** Yan Jia, Shuyi Liu, Yongqing Liu, Limin Zhai, Yifan Gong, Xiangkun Zhang

**Affiliations:** 1 Key Lab of Microwave Remote Sensing, National Space Science Center, Chinese Academy of Sciences, Beijing 100190, China; 2 School of Electronic, Electrical and Communication Engineering, University of Chinese Academy of Sciences, Beijing 100049, China

**Keywords:** CFD simulation, hydrodynamic wake, electromagnetic scattering model, SAR image

## Abstract

The paper introduces a numerical simulation method for Synthetic Aperture Radar (SAR) imaging of submerged body wakes by integrating hydrodynamics, electromagnetic scattering, and SAR imaging simulation. This work is helpful for better understanding SAR images of submerged body wakes. Among these, the hydrodynamic model consists of two sets of ocean dynamics closely related to SAR imaging, namely the wake of the submerged body and wind waves. For the wake, we simulated it using computational fluid dynamics (CFD) numerical methods. Furthermore, we compared and computed the electromagnetic scattering characteristics of wakes under various navigation parameters and sea surface conditions. Following that, based on the operational principles and imaging theory of synthetic aperture radar (SAR), we established the SAR raw echo signal of the wake. Employing a Range-Doppler (RD) algorithm, we generated simulated SAR images of the wake. The results indicate that utilizing Computational Fluid Dynamics (CFD) numerical methods enables the simulation of wake characteristics generated by the motion of a submerged body with different velocities. The backscattering features of wakes are closely associated with the relative orientation between the wake and the radar line of sight. Under specific wind speeds, the wake gets masked within the sea surface background, resulting in less discernible characteristics of the wake in SAR images. This suggests that at lower speeds of submerged body or under specific wind conditions, the detectability of the wake in SAR images significantly diminishes.

## 1. Introduction

Detecting submerged bodies is crucial for both civilian and military applications. Presently, acoustic systems like sonar serve as the primary means of submerged body detection. However, advancements in noise reduction and sound-dampening technologies have posed challenges for traditional sonar detection methods. Additionally, the multiple reflections of sound waves often result in significant localization errors for sonar systems. Consequently, the sole reliance on conventional acoustic methods no longer meets the current demands for detecting submerged bodies like submarines. As a result, there is a growing focus on non-acoustic detection methods to enhance the capability for detecting the submerged body [1,2].

Within the realm of non-acoustic observation techniques, Synthetic Aperture Radar (SAR) stands out as a pivotal means of detection, holding significant promise for diverse applications. Its notable advantages lie in the radar’s inherent high resolution, strong interference resistance, and capability for extensive and all-weather observations. However, the electromagnetic waves emitted by radar cannot directly penetrate seawater to interact with the submerged body. Instead, identification of the target can be achieved by measuring the disturbance field caused by the motion of the submerged body on the water surface (known as wakes). The features of surface wakes can further be employed for the computation of the target’s velocity, underwater depth, and direction. There has been a substantial body of research focused on SAR image simulation of surface ship wakes based on potential flow theory. Similarly, the submerged body under water, not really different from the vessel on the surface, can also excite wakes when the body is running fast at a shallow depth. There are at least four types of hydrodynamic disturbances that a submerged body can produce: the Bernoulli hump, the Kelvin wake, the turbulent wake, and internal waves [3]. These phenomena exert direct or indirect influence on the surface wakes produced by the movement of a submerged body, thereby allowing the surface characteristics of these wakes to be discerned within SAR images.

**Figure 1:**
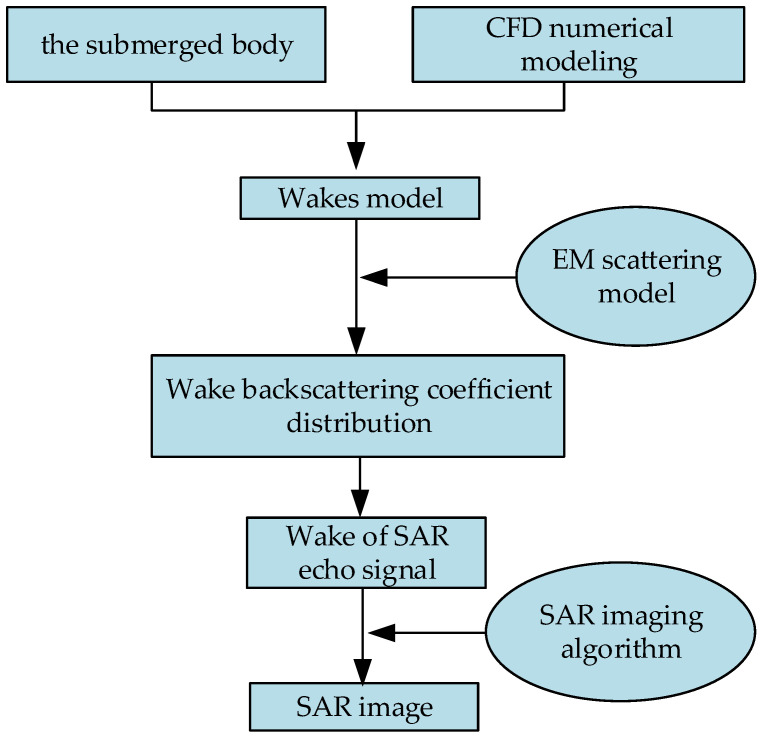
Flow diagram of SAR image of wake caused by the submerged body.

The detection outcomes of the submerged body wakes remain limited due to factors such as military confidentiality or restricted access to information. Moreover, the complexity of marine environments and dynamic conditions poses challenges in experimentally controlling real environmental parameters, thereby impeding the attainment of consistent results. A fundamental prerequisite for studying SAR imaging of wakes is the acquisition of geometric characteristics specific to surface wakes generated by the submerged body’s movements. In the realm of methodologies exploring the characteristics of wakes generated by the movement of a submerged body on the sea surface, a variety of methods are proposed by researchers, including numerical simulation techniques proposed by Michell [4], Wang [5], Egger [6], Tuck [7], and others. Computational fluid dynamics (CFD) numerical simulations are progressively emerging as a viable approach for assessing the traits of wakes under diverse parameters and conditions [8,9,10]. L. Wang et al. conducted simulations of internal waves generated by the motion of underwater objects using computational fluid dynamics (CFD) numerical modeling, and they conducted SAR image simulations based on a velocity bunching model [11]. Zilman performed simulations of Kelvin wakes in SAR images by incorporating real aperture radar (RAR) and specific SAR imaging mechanisms [12]. Liu and Jin conducted SAR image simulations of dynamic submerged body wakes [13]. Zhang conducted simulations of internal wave wakes generated by underwater moving objects, using an interferometric SAR imaging process, in [14]. However, the mentioned study did not initiate SAR imaging simulations from the establishment of SAR raw echo signals for wakes. In this study, our objective is to simulate the received radar echo signals and subsequently focus the SAR raw signals of wakes using SAR imaging algorithms to generate SAR images of wakes. The main flowchart depicting the process of wake SAR imaging is illustrated in Figure 1.

The remaining sections of this paper are structured as follows: Section 2 provides a detailed description of the geometric model for randomly rough sea surfaces and the model for submerged body wakes. Section 3 presents the SAR imaging method based on echo signals of wakes, discussing and analyzing the electromagnetic scattering characteristics of wakes under different conditions and their corresponding SAR images. Finally, Section 4 presents the conclusions and prospects drawn from this study.

## 2. Modeling Submerged Body Wakes in Complex Sea Surface Backgrounds

### 2.1. The Fluid Dynamics Model of Submerged Body Wakes

**Figure 2:**
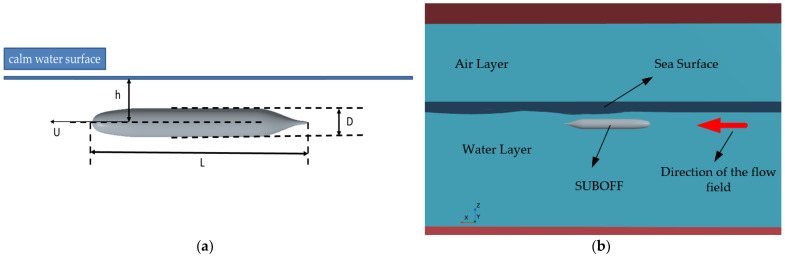
Simulation structure. (**a**) SUBOFF model. (**b**) Demonstration of CFD simulation.

As shown in Figure 2a, the selected form of the submerged body in this study is the DARPA SUBOFF hull form, as described by Groves et al. [15,16]. The SUBOFF is a submarine model specifically designed by the U.S. Defense Advanced Research Projects Agency (DARPA) to establish a Computational Fluid Dynamics (CFD) analysis software validation database for submarines. The current work only considers the bare hull, consisting primarily of the bow, parallel midsection, and stern, excluding the rudder and stern appendages. The bare hull model is sized down by a factor of 24 from the actual dimensions of the SUBOFF submarine. The SUBOFF model length L=4.356 m, and the maximum diameter D=0.508 m. The model travels at a constant speed U underwater at a depth of h, where h represents the distance between the plane $z_{0}=0$ and the main axis of the vessel. In this paper, the relative motion of the SUBOFF model is simulated by setting the water flow velocities to 3 m/s and 5 m/s within the CFD simulation.

The CFD numerical simulation of the wake is based on the Reynolds-Averaged Navier–Stokes (RANS) equations [17,18], implemented using the STAR-CCM+ (version 2302 18.02.008-R8) software platform. The computer’s hardware configuration comprises Windows 10 as the operating system, an Intel(R) Core(TM) i5-7300 CPU @ 2.50 GHz (Intel, Santa Clara, CA, USA) for the central processing unit (CPU), and an NVIDIA GeForce GTX 1050 Ti (NVIDIA, Santa Clara, CA, USA) for the graphics processing unit (GPU). The finite volume method discretizes the governing equations, employing the SIMPLE algorithm for solving the pressure–velocity coupling terms. The Realizable k-ε turbulence model is employed, while the Volume of Fluid (VOF) method captures the free surface. Grid refinement is performed at the bow, stern, and the free liquid surface of the submerged body. Taking the longitudinal profile of the submarine as a symmetrical plane, the computational model was set as symmetrical to optimize the utilization of computational resources. The CFD simulation of the submerged body is depicted in Figure 2b, where the simulation domain is divided into the water and air layers. The upper layer represents the air, while the lower layer represents the water. Under low-wind conditions, the free surface between the water and air is typically extracted to calculate the electromagnetic scattering field. The red arrows indicate the direction of water flow, aligned with the positive direction of the *x*-axis. Numerical calculations simulated a total of two states as shown in Table 1.

**Figure 3:**
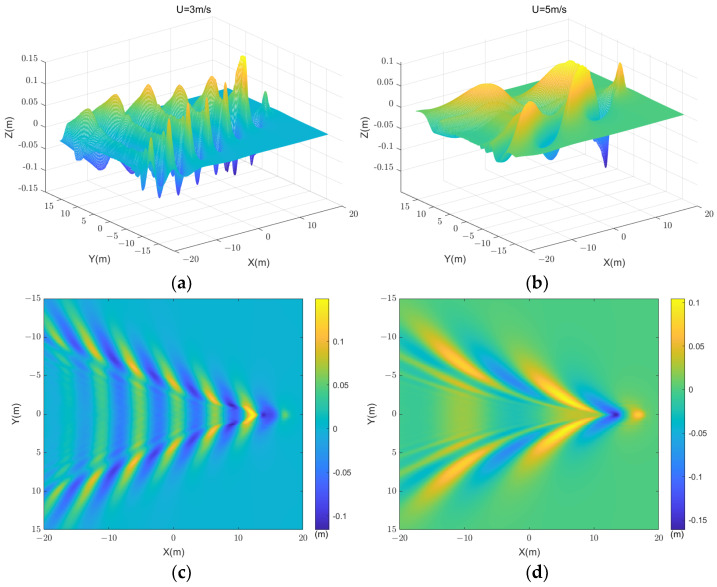
The geometric features of wakes at the free surface for SUBOFF moving at different velocities. (**a**,**c**) U = 3 m/s; (**b**,**d**) U = 5 m/s.

When the submerged body moves close to a calm water surface, it induces wave formations resembling the classical Kelvin waves on the tranquil water surface. These waves consist of a blend of different elements like the Bernoulli peak and turbulent wakes, which are not individually addressed in this study. In comparison to the linear wave potential theory, CFD numerical simulations not only provide highly accurate numerical solutions but are also versatile in handling various boundary conditions and geometries. Regrettably, due to computational constraints, the scope of simulated wake scenes in this study is limited. Reconstructing the wakes obtained through the CFD numerical method is essential for subsequent electromagnetic scattering analysis of wakes. By interpolating the wake data extracted from the CFD grid, the information is restructured into a collection of 0.1 m × 0.1 m elemental data. Figure 3 illustrates the two-dimensional and three-dimensional images of wakes at different speeds for the same depth.

**Figure 4:**
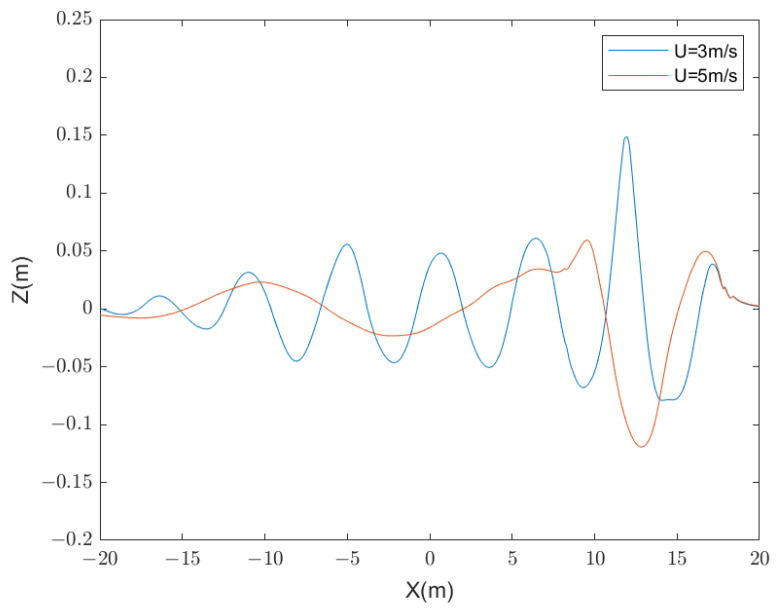
The comparison of wave heights at the centerline of the wakes.

It is evident that when the submerged body navigates near the water surface, the wake pattern behind it forms a wave system similar to the Kelvin wake pattern generated by surface vessels. At the leading edge of the wake wave-front of the submerged body isa wave peak, known as the Bernoulli peak. Additionally, this system can be classified into transverse and spreading waves based on the direction of movement. The velocity of the submersible model can be represented by Froude number $Fr=U/\sqrt{gL}$. It is notable that in the surface wake at Fr=0.46, both the divergent and transverse wave are discernible in the image. Conversely, at Fr=0.77, the wake predominantly displays divergent wave characteristics. Some studies have shown that when Fr=0.46, at low or moderately high Froude numbers, transverse waves are clearly visible. When Fr=0.77, the transverse wave basically disappears. Figure 4 delineates the wave height profiles along the centerline of the surface wake at these two distinct speeds.

From Figure 4, it can be observed that as the velocity increases, the amplitude difference between the wave peaks and troughs also increases. According to wave analysis theory, within Kelvin wakes, the wavelength is solely dependent on the speed of the submerged body. As the speed increases, the wavelength also increases [19,20]. The theoretical wavelengths corresponding to different speeds are:

(1) $$\lambda =\frac{2\pi }{g}U^{2},$$

Table 2 presents the comparison between simulated and theoretical wavelengths at two different speeds. The numerical simulation results are in close agreement with the theoretical wavelengths, demonstrating the accuracy of the submerged body wake simulation.

### 2.2. Modeling of Randomly Rough Sea Surfaces

**Figure 5:**
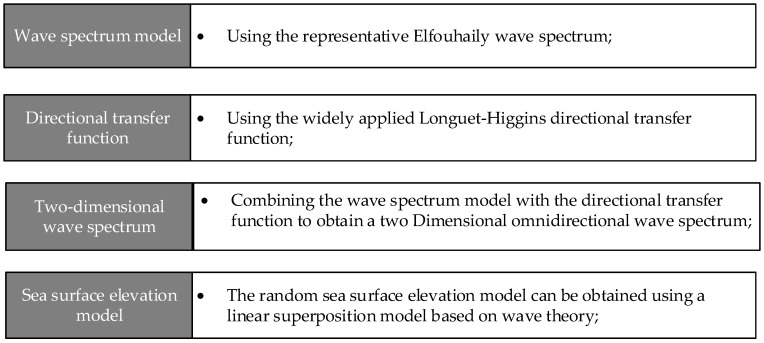
Random two-dimensional sea surface generation diagram.

Figure 5 depicts a schematic for generating randomly rough sea surfaces. This study utilizes the representative Elfouhaily wave spectrum in conjunction with the one-sided Longuet-Higgins directional transfer function to derive a two-dimensional omnidirectional spectrum.

The Elfouhaily spectrum is extensively employed in sea surface modeling due to its advantage of being relatively straightforward to understand through empirical formulas and its closer resemblance to actual sea surfaces [21]. It is represented by Equation (2):

(2) $$\Psi (k)=\frac{1}{k^{3}}\left[B_{L}(k)+B_{S}(k)\right],$$

where, $B_{L}(k)$ and $B_{S}(k)$ represent the high frequency curvature spectrum and the low frequency curvature spectrum, respectively, and k is the sea surface wave number, and Ψ(k) is the wave spectrum as a function of angular spatial frequency. The spectrum Ψ(k) alone is inadequate to comprehensively characterize wave propagation in a two-dimensional space. Therefore, the Longuet-Higgins directional transfer function in unilateral form is used to describe the two-dimensional sea spectrum [22]. The formula is described by Equation (3):

(3) $$D(k,\theta )=\frac{\Gamma (S+1)}{\left[\Gamma (S+0.5)2\sqrt{\pi }\right]}{cos}^{2S}\left(\frac{\theta -\theta_{w}}{2}\right),$$

In this equation, Γ(·) represents the gamma function, and parameter S is dependent on wavenumber k, and the directionality of the wave increases as the parameter S increases. We retain S as a constant value. θ represents the direction of wave propagation, while $\theta_{w}$ signifies the mean wind direction. Combining the wave spectrum with the directional transfer function, the two-dimensional wave spectrum can be represented as:

(4) $$S_{E-LH}(k)=\frac{1}{k}\Psi (k)D\left(k,\theta -\theta_{w}\right),$$

**Figure 6:**
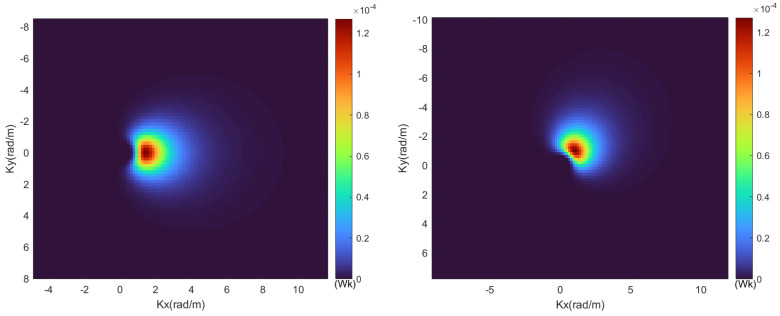
Two-dimensional sea spectral images under different wind directions.

Figure 6 shows the two-dimensional wave spectrum under wind speed $V_{w}=2\;m/s$ at a height of 10 m above the sea surface and different wind angle conditions. From Figure 6, it is evident that as the wind angle $\theta_{w}$ changes, the distribution angle of the two-dimensional wave spectrum on the sea surface also changes, following the same direction as the variation in the wind angle. Therefore, the two-dimensional wave spectrum can reflect the energy distribution of two-dimensional waves under different wind directions, such as downwind and upwind conditions.

If deep water is assumed and non-linear effects are neglected, the wind-driven surface can be generated by a linear superposition of plane progressive waves (PPWs) for all possible frequencies and directions [23]:

(5) $$Z_{\mathrm{sea}\;}(x,y,t)=\sum_{i}\sum_{j}A_{ij}cos\left[k_{i}\left(xcos\theta_{j}+ysin\theta_{j}\right)-\omega_{i}t+\in_{ij}\right]$$

**Figure 7:**
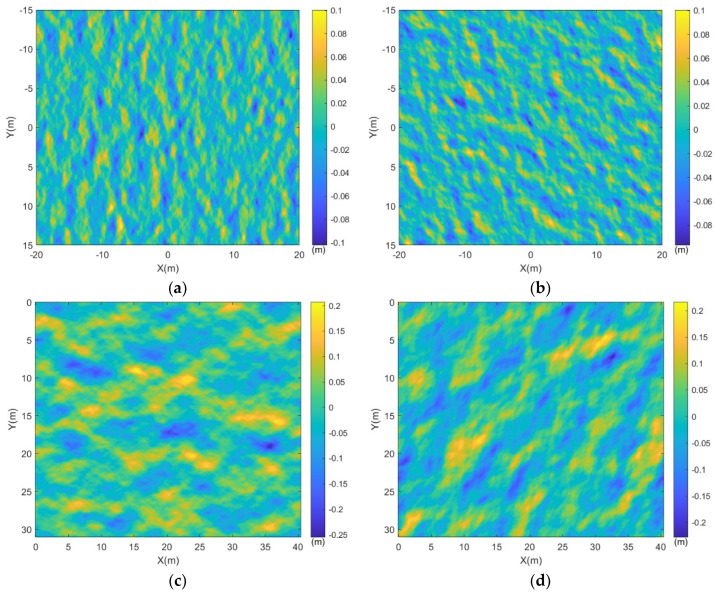
Random sea surfaces under different wind velocities and directions. (**a**) $\theta_{w}=0{}^{\circ}$, $V_{w}=2\;m/s$; (**b**) $\theta_{w}=45{}^{\circ}$, $V_{w}=2\;m/s$; (**c**) $\theta_{w}=90{}^{\circ}$, $V_{w}=3\;m/s$; (**d**) $\theta_{w}=135{}^{\circ}$, $V_{w}=3\;m/s$.

$\in_{ij}$ represents the initial phase; $k_{i}$, $\omega_{i}$ and $\theta_{j}$ represent separately wavenumber, angular frequency, and plane progressive wave (PPW) propagation angle. $A_{ij}$ can be expressed as:

(6) $$A_{ij}=\sqrt{2\Psi (k_{i})D\left(k_{i},\theta_{j}\right)\Delta k_{i}\Delta \theta_{j}}$$

where ∆k and ∆θ represent the sampling intervals for wavenumber and propagation angle. Figure 7 illustrates the randomly generated two-dimensional rough sea surfaces under different conditions. To facilitate subsequent electromagnetic scattering calculations combined with wake modeling, the sea surface dimensions are maintained consistent with the dimensions of the submerged body wake described in the previous section.

The comparison between the four images in Figure 7 reveals that the propagation direction of the sea waves is strictly controlled by the directional transfer function in the sea spectrum. As the wind direction changes, the sea waves also exhibit corresponding variations. It can be observed that with an increase in the wind speed $V_{w}$ above the sea surface, the undulation of the sea waves also increases. Moreover, within the same field of view of the sea surface, there is a tendency for the wavelength scale of the waves to enlarge.

**Figure 8:**
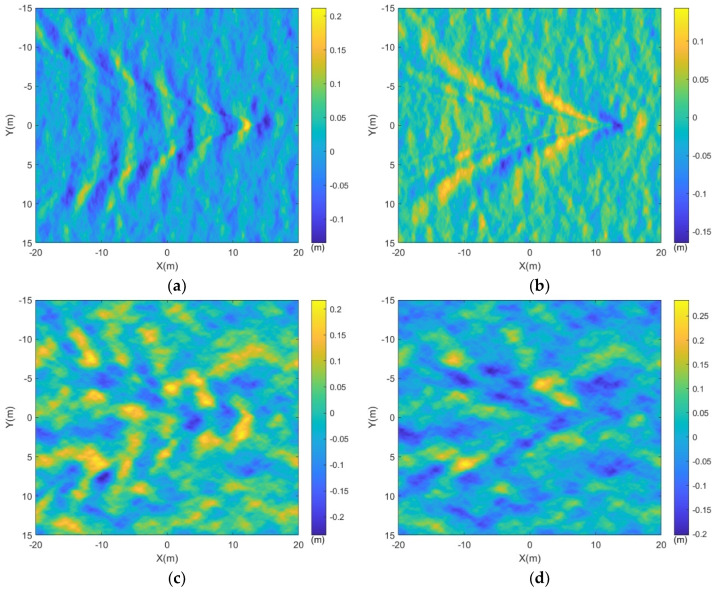
The wind-driven sea surface and the submarine wake. (**a**) $V_{w}=2\;m/s$, U=3 m/s; (**b**) $V_{w}=2\;m/s$, U=5 m/s; (**c**) $V_{w}=3\;m/s$, U=3 m/s; (**d**) $V_{w}=3\;m/s$, U=5 m/s.

The wake obtained from the CFD numerical simulation in the previous section was superimposed onto the sea surface under different wind speed parameters depicted in Figure 7a,c, resulting in the representation of the target wake with a wind-driven sea surface, as illustrated in Figure 8.

Comparing Figure 8a with Figure 8c, and Figure 8b with Figure 8d, it is observed that when the wind speed over the sea surface is 2 m/s, the characteristics of the submerged body wakes are relatively pronounced against the sea surface background. However, as the wind speed increases, the roughness of the sea surface intensifies, and the wake features within the sea surface become obscured within the background, rendering the wake characteristics less discernible.

## 3. Electromagnetic Modeling of Submerged Body Wakes

**Figure 9:**
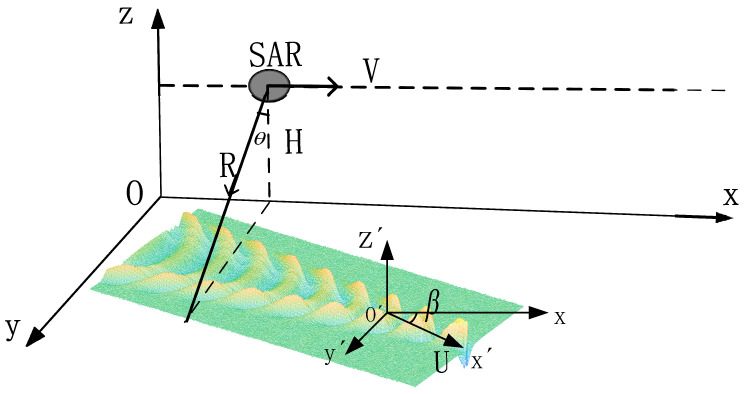
The geometry of the SAR wakes imaging. The orthogonal coordinate systems *Oxyz* are fixed in the Earth, whereas the coordinate system *O*’*x*’*y*’*z*’ is fixed in a moving submarine. The planes Oxy and *O*’*x*’*y*’ coincide with the plane of the sea surface; the axes *Oz* and *O*’*z*’ are perpendicular to this plane and are directed upward. The vector of the velocity of the radar platform V is parallel to the plane *Oxy* and is directed along the axis *Ox*.

Figure 9 shows the geometry of the SAR wakes imaging. *H* is the flight height. The slant range distance *R* of the SAR platform is given by R=H/cos⁡θ, where *θ* is the radar incident angle. The velocity U of submarine is directed along the axis *O*’*x*’. The axis *Ox* is constituted with the axis *O*’*x*’ with angle β. At β=0°, the direction of motion of the SAR platform is parallel to the direction of movement of the submarine; at β=90°, the SAR platform’s motion direction is vertical to the direction of movement of the submarine.

Before the SAR signature of the wake, EM scattering distributions need to be computed. When calculating the electromagnetic scattering coefficient of wakes, there are two main scattering mechanisms: Bragg scattering and non-Bragg scattering (from breaking waves and specular reflections) [24]. SAR operates primarily in the centimeter to decimeter wavelength range with moderate incidence angles ranging from 20° to 70° for VV polarization (20° to 60° for HH polarization), which is directly related to Bragg scattering [25,26]. This study focuses specifically on the Bragg scattering region, which is determined by the sea surface roughness corresponding to the wavelength of the radar signal. Furthermore, the decision to exclusively use VV and HH polarizations was due to the better clarity of the imaged wakes in co-polarization SAR.

The utilization of the two-scale model (TSM) in this study is grounded in resonant Bragg scattering theory, offering a judicious balance between computational efficiency and accuracy in approximating scattering phenomena [25,27,28]. The solution for Bragg scattering in the ensemble-averaged normalized radar cross-section (NRCS) with VV and HH polarizations is formulated as follows [29]:

(7) $$\sigma_{0}(x,y)=8\pi k_{e}^{4}{cos}^{4}\theta_{l}W\left(k_{Bx},k_{By}\right)|T{|}^{2}$$

(8) $$T={\left[\frac{sin\left(\theta +s_{p}\right)coss_{p}}{sin\theta_{l}}\right]}^{2}b_{PP}\left(\theta_{l}\right)+{\left(\frac{sins_{n}}{sin\theta_{l}}\right)}^{2}b_{QQ}\left(\theta_{l}\right)$$

where $k_{e}=2\pi /\lambda$ represents radar electromagnetic wavenumber, λ is the wavelength of the radar signal, $\theta_{l}$ is the radar’s local incidence angle. W (∙) is the two-dimensional wavenumber spectral density of sea surface roughness. $k_{Bx}=2k_{e}sin\left(\theta +s_{p}\right)coss_{p}$, and $k_{By}=2k_{e}\cos\left(\theta +s_{p}\right)\sin s_{n}$ are the resonance Bragg wavenumbers pertaining to the tilted surface of large-scale gravity waves. T (⋅) is the complex scattering function which controls polarization of the radar signal and depends on the relative dielectric constant e of the seawater. The subscripts *PP* and *QQ* in (8) denote the vertical–vertical (VV) and horizontal–horizontal (HH) polarizations (or vice versa), $b_{VV}=\varepsilon^{2}\left(1+{sin}^{2}\theta_{l}\right)/{\left(\varepsilon cos\theta_{l}+\sqrt{\varepsilon }\right)}^{2}$, $b_{HH}=\varepsilon /{\left(cos\theta_{l}+\sqrt{\varepsilon }\right)}^{2}$, and ε=48−35i for X-band; ε=60−36i for C-band; and ε=72−59i for L-band [30].

The local incident angle $\theta_{l}$ of electromagnetic waves on each sloping element can be derived from the element’s slope. It can be expressed as:

(9) $$\theta_{l}=arccos\left[cos\left(\theta -s_{p}\right)coss_{n}\right]$$

The local wave slopes $s_{n}$ and $s_{p}$ in (9) represent the slope components parallel and perpendicular to the radar sight direction, respectively, which can be represented by the derivative of the wave height on a slightly inclined sea surface:

(10) $$s_{n}=\frac{\partial Z}{\partial x},s_{p}=\frac{\partial Z}{\partial y}$$

**Figure 10:**
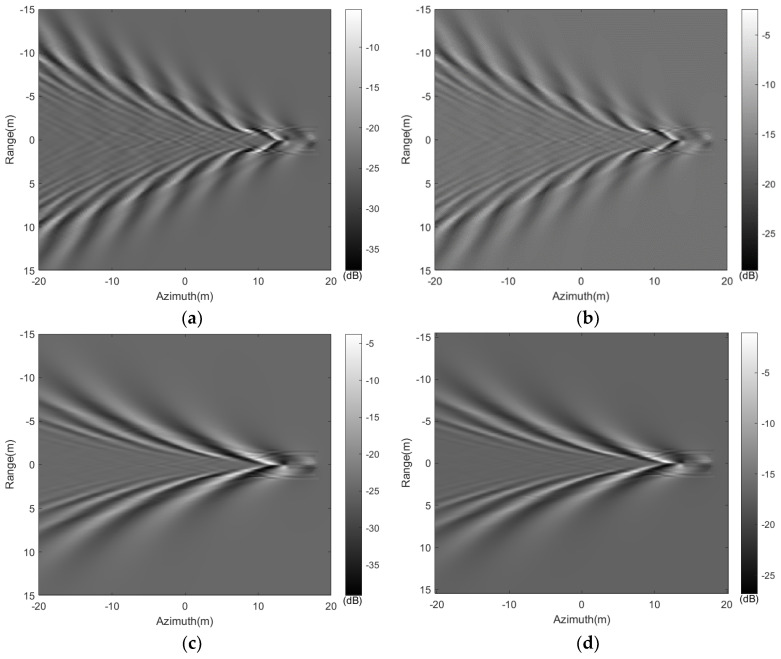

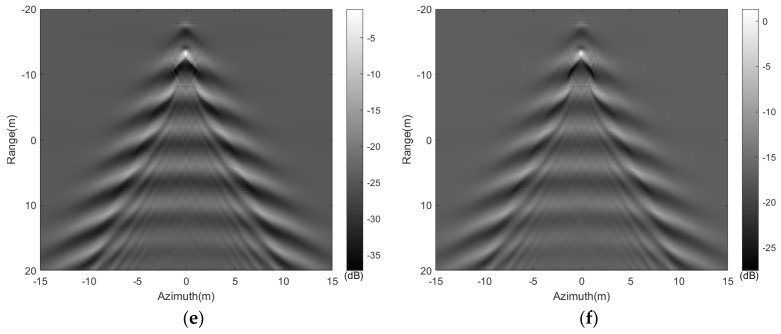
Simulated NRCS images of the wakes based on Figure 3. (**a**): HH polarization, U = 3 m/s, β=0°; (**b**) VV polarization, U = 3 m/s, β=0°; (**c**): HH polarization, U = 5 m/s, β=0°; (**d**): VV polarization, U = 5 m/s, β=0°; (**e**): HH polarization, U = 3 m/s, β=90°; (**f**): VV polarization, U = 3 m/s, β=90°.

Using the aforementioned electromagnetic scattering model, the backscatter coefficient distribution of the wake shown in Figure 3 on a flat sea surface was initially considered. At a radar frequency of 5.3 GHz (C-band) and an incidence angle of 30 degrees, Figure 10 computed the backscatter coefficient distribution of the wakes. From Figure 10, it is apparent that the backscatter distribution image of the wake, calculated using the mentioned scattering model, displays noticeable characteristics of brightness and darkness. HH polarization differs primarily in amplitude from VV polarization, while their pattern features are similar. Figure 10a–d shows the backscatter distribution diagram of wakes at different speeds when the target movement direction is perpendicular to the radar line of sight (β = 0°), while Figure 10e,f shows the backscattering coefficient distribution of the wake when the target movement direction is parallel to the radar line of sight (β = 90°). Comparing Figure 10a,b,e,f, it is noticeable that in the scattering images of (a,b), the transverse wave component is not observed, while it can be seen in images (e,f). In subfigures (a,b), the scattering coefficient of the transverse waves in the wake, whose wave propagation vector is perpendicular to the radar line of sight, exhibits a smaller amplitude and is not prominently visible in the backscatter coefficient distribution image. Conversely, in subfigures (e,f), the transverse waves within the wake, having their wave propagation vector parallel to the radar line of sight, display a higher backscattering coefficient amplitude, thus appearing brighter in the backscatter coefficient distribution image.

**Figure 11:**
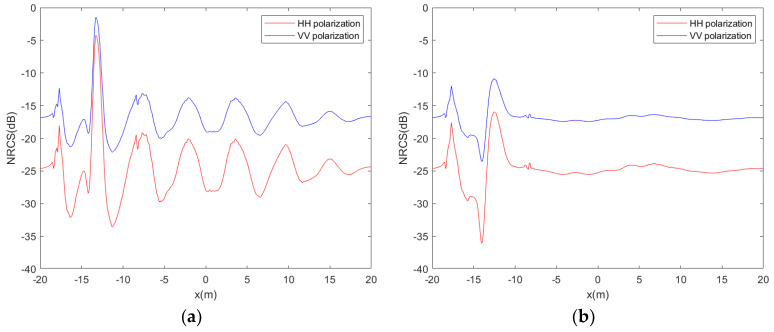
The NRCS of the centerline of the wakes for different polarizations; (**a**) U = 3 m/s; (**b**) U = 5 m/s.

Figure 11 depicts the backscattering coefficients along the centerline of the target wake at β = 90°. This illustration provides a clearer demonstration of the discrepancies in backscattering coefficients between HH and VV polarizations. A comparative analysis indicates that the normalized radar cross-section (NRCS) in VV polarization exceeds that of HH polarization. However, the disparity in NRCS values between peaks and troughs is more pronounced in HH polarization. The comparison between the two figures also reveals the absence of transverse waves in the wake at a speed of 5 m/s.

**Figure 12:**
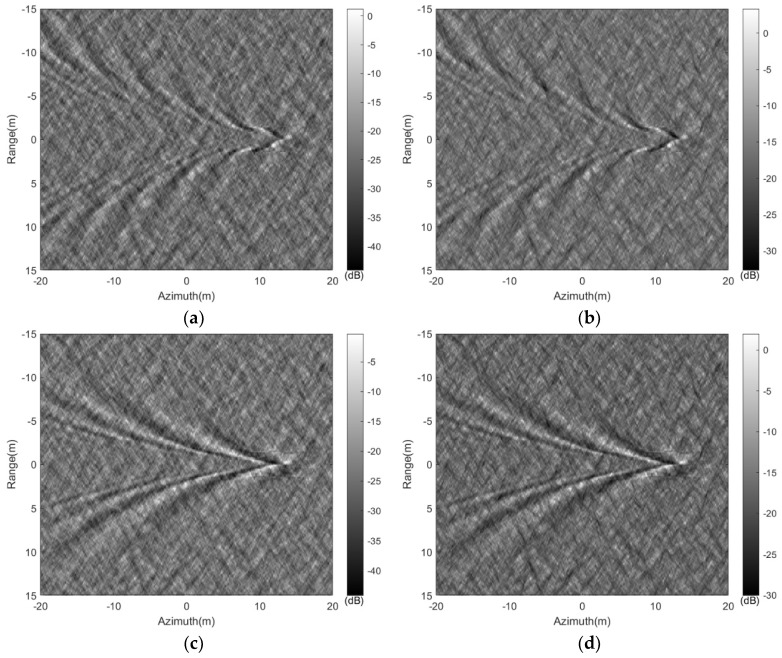
The backscattering coefficient distribution of wakes under wind-driven sea surface. (**a**): HH polarization, U = 3 m/s; (**b**) VV polarization, U = 3 m/s; (**c**): HH polarization, U = 5 m/s; (**d**): VV polarization, U = 5 m/s.

Further, Figure 12 depicts the backscattering coefficient distribution of wakes under wind-driven sea surface background at $V_{w}=2\;m/s$ in Figure 8. The image distinctly displays the variation in wake backscattering coefficient distribution caused by the change in roughness due to the wind-driven sea surface. A comparison between the wakes of two different speeds in Figure 12 reveals that longer wavelength wakes exhibit relatively clear brightness and darkness characteristics in the image, while shorter wavelength wakes demonstrate less apparent brightness and darkness features under the wind-driven sea surface background.

## 4. SAR Imaging Simulation of the Wake over the Sea Surface

At present, scholars both domestically and internationally categorize methods for simulating SAR imaging in maritime environments into two main types: those based on SAR image features and those based on echo signal simulations. We use the SAR imaging simulation method based on echo signals. The simulation method, relying on echo signals, involves processing the model’s echo signals using imaging algorithms to generate simulated SAR images. While this method accurately mirrors the characteristics of SAR systems and offers robust real-time capabilities, it demands significant computational resources for echo calculations. We study the SAR image simulation of ocean scenes at the echo signal level in this section. The simulation process includes the generation of the raw signal, and SAR imaging algorithm.

### 4.1. SAR Imaging Processing of Wake over the Sea Surface Based on SAR Echo Signals

Applying SAR imaging principles and simulation techniques to submerged body wakes involves simulating SAR raw echo signals. Typically, the signals emitted by SAR are linear frequency-modulated pulses and can be represented as follows:

(11) $$s_{pul}\left(t\right)=\sum_{n=-\infty }^{+\infty }rect\left(\frac{t-n\cdot PRT}{T_{r}}\right)\exp\left\{j\left[2\pi f_{c}\left(t-n\cdot PRT\right)+\pi K_{r}{\left(t-n\cdot PRT\right)}^{2}\right]\right\}$$

In (11), rect(·) is the range signal envelope, which is an approximately rectangular window, and $f_{c}$ is the radar carrier frequency and $K_{r}$ is the range frequency modulation (FM) rate, PRT is the pulse repetition time, and $T_{r}$ represents the pulse duration. SAR uses the Doppler history of signals to achieve fine azimuth resolution. Based on the “stop-and-go” assumption, we can assume that wakes are stationary. The platform’s minimum slant range is denoted as $R_{0}$, with a platform speed of V and a radar incident angle of θ. During the computation of electromagnetic scattering from the wakes, the wakes are divided into a collection of many facets; for each facet, the point target echo signal model can be applied. Thus, following the echo signal model for the point target, the echo signal for each facet can be expressed as:

(12) $$Sr=A_{0}w_{r}\left(\tau -\frac{2R(\eta )}{c}\right)w_{a}\left(\eta -\eta_{c}\right)exp\left\{j\pi \left[K_{r}{\left(\tau -\frac{2R(\eta )}{c}\right)}^{2}-\frac{4f_{c}R(\eta )}{\lambda }\right]\right\}$$

τ is the range time, η is azimuth time, $w_{r}$ and $w_{a}$ correspond to the range envelope and azimuth envelope, R(η) is the slant range from each facet to the radar. $A_{0}$ is linked to SAR system parameters and the backscattering coefficient, which can be expressed by the radar equation:

(13) $$A_{0}=\sqrt{\frac{P_{t}G^{2}\lambda^{2}(∆A)}{(4\pi {)}^{3}R(k{)}^{4}}\sigma^{0}}$$

where ∆A is the area of each facet, $\sigma^{0}$ is the backscattering coefficient for each facet, $P_{t}$ is average transmitted power, while G is the antenna gain.

**Figure 13:**
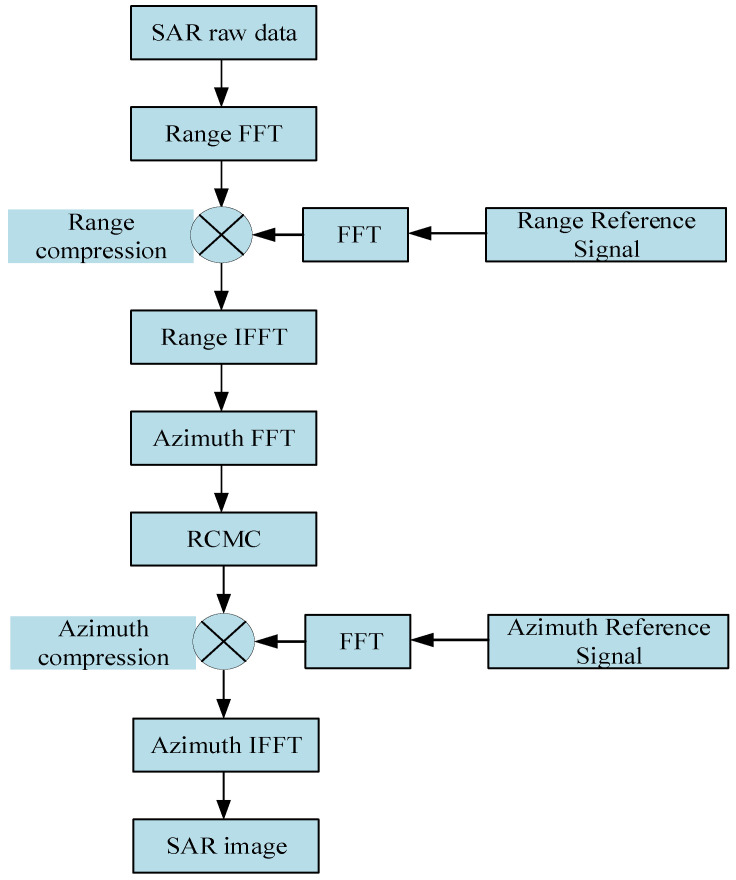
Flowchart of the RDA.

The sea wake is considered a compilation of surface elements featuring numerous independent scattering points. The computation of the echo signal from the sea surface wake is achieved by applying the point target echo signal model, as described in Equation (12). Following this, the Range-Doppler algorithm (RDA) is employed to simulate the sea wake in SAR images [31]. A detailed illustration of the Range-Doppler algorithm (RDA) process is provided in Figure 13.

The echo signal in (12) is the SAR raw signal without focusing. The implementation process of the RD algorithm is as follows:

1. Upon conducting the fast Fourier transform (FFT) along the range direction, the SAR raw echo signal in the frequency domain along the range dimension can be represented as:
(14) $$S_{0}\left(f_{\tau },\eta \right)={FFT}_{r}\left(s_{0}(\tau ,\eta )\right)=A_{0}W_{r}\left(f_{\tau }\right)w_{a}\left(\eta -\eta_{c}\right)exp\left\{-j\pi \left[\frac{{\left(f_{\tau }\right)}^{2}}{K_{r}}+\frac{4f_{c}R(\eta )}{\lambda }\right]\right\}$$
2. The SAR raw echo signal is multiplied by the range-matched filter $H_{r}\left(f_{\tau }\right)$ in the range frequency domain to eliminate the second-order phase term related to fast time τ. The expression for the range-matched filter is:
(15) $$H_{r}\left(f_{\tau }\right)=rect\left(\frac{f_{\tau }}{\left|K_{r}\right|T_{r}}\right)exp\left(+j\pi \frac{{\left(f_{\tau }\right)}^{2}}{K_{r}}\right)$$
  The echo signal after the range compression is expressed as:
  (16) $$s_{rc}\left(\tau ,\eta \right)={IFFT}_{r}\left\{S_{0}\left(f_{\tau },\eta \right)H_{r}\left(f_{\tau }\right)\right\}$$
3. Changes in the instantaneous slant range lead to range cell migration, requiring correction. Range cell migration correction (RCMC) is implemented post-range compression and prior to azimuth compression. RCMC is commonly implemented within the Range-Doppler domain. The echo signal and the slant range formula in the Range-Doppler (RD) domain are obtained by azimuthal FFT and are given by:
(17) $$S_{rc}\left(\tau ,f_{\eta }\right)={FFT}_{a}\left(s_{rc}\left(\tau ,\eta \right)\right)$$
  The amount of RCM to correct is given by:
  (18) $$∆R\left(f_{\eta }\right)=\frac{\lambda^{2}R_{0}f_{\eta }^{2}}{8V^{2}}$$
  The Sinc interpolation operation can be used for the range cell migration correction (RCMC), Assuming the RCMC interpolation is applied accurately, the signal can be expressed as:
  (19) $$S_{rcmc}\left(\tau ,f_{\eta }\right)=A_{0}w_{r}\left(\tau -\frac{2R_{0}}{c}\right)w_{a}\left(f_{\eta }-f_{\eta c}\right)exp\left\{-j\pi \left[\frac{{\left(f_{\eta }\right)}^{2}}{K_{a}}+\frac{4{\;f_{c}R}_{0})}{\lambda }\right]\right\}$$
  In (19), the range envelope $w_{r}$ is now independent of azimuth frequency, indicating that the RCM has been corrected.
4. An azimuth matched filter $H_{az}\left(f_{\eta }\right)$ in the Range-Doppler domain is used to achieve azimuth compression and can be given by
(20) $$H_{az}\left(f_{\eta }\right)=\exp\left(-j\pi \frac{R\lambda }{2V^{2}}f_{\eta }^{2}\right)$$
  The 2D time domain complex amplitude of compressed signal $s_{ac}\left(\tau ,\eta \right)$ is obtained after inverse fast Fourier transform (IFFT) along the azimuth direction.
  (21) $$s_{ac}\left(\tau ,\eta \right)={IFFT}_{a}\left\{S_{rcmc}\left(\tau ,f_{\eta }\right)H_{az}\left(f_{\eta }\right)\right\}$$
  The above is the process for the actual imaging for wakes over a sea surface.

### 4.2. Results and Discussion

The previous section outlined the division of the wake scene into many facets, with each facet having a dimension of 0.1 m × 0.1 m. For the 0.1 m × 0.1 m facets, lower radar resolution can impact wake detection, while higher resolution increases computational load. To maintain wake imaging without excessive computational burden, a radar resolution of approximately 0.25 m is chosen. The specific SAR imaging simulation parameters are presented in Table 3.

**Figure 14:**
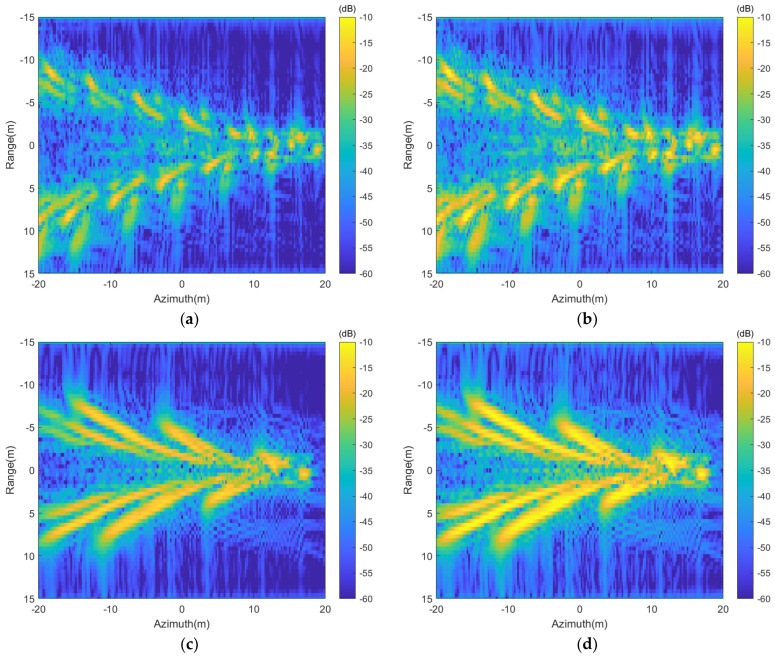

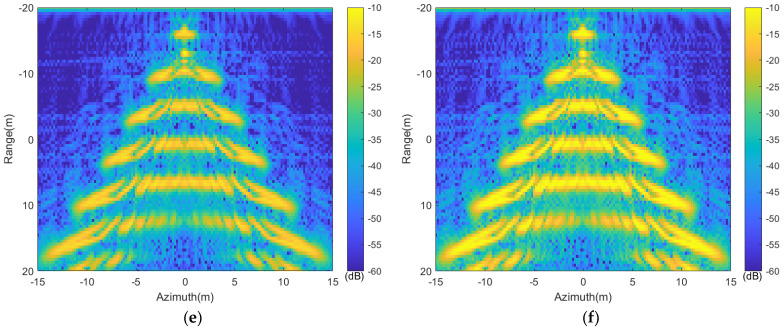
Simulated SAR image of the wake over flat surface: (**a**): HH polarization, U = 3 m/s, β=0°; (**b**): VV polarization, U = 3 m/s, β=0°; (**c**): HH polarization, U = 5 m/s, β=0°; (**d**): VV polarization, U = 5 m/s, β=0°; (**e**): HH polarization, U = 3 m/s, β=90°; (**f**): VV polarization, U = 3 m/s, β=90°.

To highlight the typical features caused solely by the moving wake, the simulated SAR images of the wakes model in Figure 3 are presented in Figure 14 in ground-range display.

It is clear that under the resolution condition of 0.25 m, the target wakes display distinct geometric features in the SAR image. As shown in Figure 14a–d, when the target moves parallel to the direction of the radar platform (β = 0°), the transverse wave component of the submerged body wake propagates perpendicular to the radar line of sight, contributing little to the radar scattering echo. Hence, only the divergent wave component can be observed in the SAR image. Figure 14e,f shows the SAR image of the wake when the target movement direction is parallel to the radar line of sight (β = 90°). In Figure 14e,f, the transverse waves within the wake have their wave propagation vector parallel to the radar line of sight, thus appearing brighter in the SAR image. As the submerged body’s speed increases, its corresponding divergent wave’s wavelength increases, appearing more pronounced in the SAR image. The variation between SAR images of different polarizations operating at identical speeds primarily presents itself through differences in brightness. In contrast to HH polarization, VV polarization displays stronger echo signals, thereby creating a notably brighter appearance within the SAR image.

**Figure 15:**
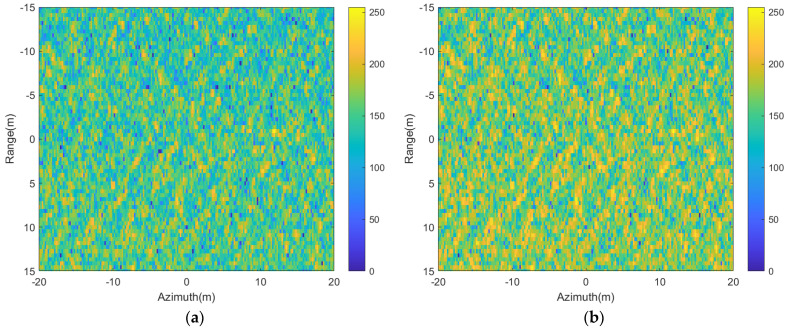

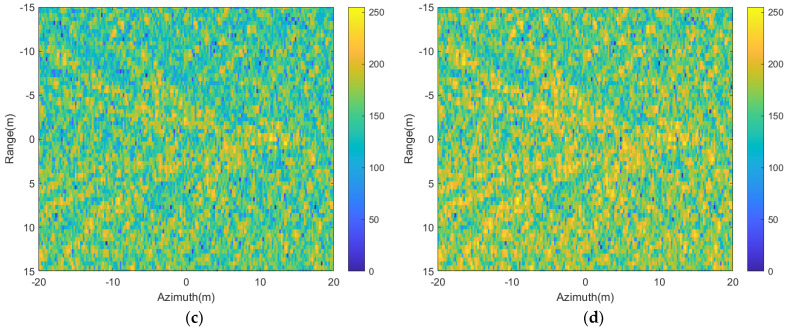
The SAR images of wakes under wind-driven sea surface. (**a**): HH polarization, U = 3 m/s; (**b**): VV polarization, U = 3 m/s; (**c**): HH polarization, U = 5 m/s; (**d**): VV polarization, U = 5 m/s.

The wakes in Figure 8a,b are superimposed on the sea surface with a cross wind of 2 m/s; the simulated SAR images of the wakes in Figure 8a,b are presented in Figure 15.

It is evident that when the submerged body travels at a speed of 3 m/s with a surface wind speed of 2 m/s, the wake features are essentially invisible in the SAR image. However, when the submerged body’s speed increases to 5 m/s under the same conditions, the characteristics of the wake become observable in the SAR image. Additionally, one can note that the intensity of SAR images in VV polarization tends to be higher than that in HH polarization, resulting in increased brightness within the image. Upon comparing the wake characteristics across varying polarizations, as illustrated in Figure 15c,d, the SAR image in HH polarization distinctly displays clearer wake features when contrasted with the VV polarization image.

## 5. Conclusions

To access the possibility of detecting the wake of a submerged body by SAR, we have integrated both hydrodynamic and electromagnetic modeling to simulate the wavemaking, backscattering, and SAR imaging process for a range of sailing parameters and ambient conditions. First, we employed the linear filtering method, CFD numerical simulation, to simulate the elevation of the wind waves and wakes of the submerged body. Then, upon utilizing the established SAR imaging platform, the composite surface scattering model facilitated the computation of the wake’s backscattering field. Subsequently, an analysis of the wake’s backscattering characteristics was conducted. Furthermore, we applied the Range-Doppler algorithm (RDA) for pulse compression and range cell migration correction (RCMC) to generate SAR images based on the raw data.

The CFD simulation results demonstrate the feasibility of simulating the wake generated by the underwater movement of a submarine model. The principal components of the wave vector within the wake vary with different velocities. At lower Froude numbers, both the divergent and transverse waves of the wake are observable. However, as the Froude number increases, the transverse wave diminishes and eventually disappears. The electromagnetic scattering properties of the wake reveal distinct differences between HH and VV polarizations, primarily in terms of amplitude. While their pattern features remain similar, the normalized radar cross-section (NRCS) in VV polarization exceeds that of HH polarization. For the SAR image of wakes, when wind speeds are elevated over the sea, the sea surface background tends to obscure the wakes generated by submerged bodies. Consequently, these wakes are rendered indiscernible in SAR images during periods of high wind. However, increased velocities of submerged bodies lead to pronounced surface wake features. Under conditions of low wind speed, it becomes feasible to observe these distinctive wake characteristics within SAR images.

There is still much work to be done in this field. In the future, we aim to delve deeper into SAR imaging simulations encompassing a wider array of submerged body wakes. Additionally, we will examine how orbital velocity affects facet displacement in SAR images when considering the VB effect. Furthermore, our investigation will encompass diverse conditions, including studying the influence of swell on submerged body wake SAR imaging outcomes.

## Figures and Tables

**Table 1:** The parameters involved in CFD numerical simulation.

Parameter	Units	I	II
Depth (h)	m	h = 1.1D	
SUBOFF velocity (*U*)	m/s	3	5
Wake scene size	m × m	30 × 40	

**Table 2:** Comparison of wakes at different speeds.

Velocity (m/s)	Trough (m)	Peak (m)	Theoretical Wavelength (m)	Actual Wavelength (m)
3	−0.11	0.138	5.764	5.75
5	−0.161	0.077	16.01	17.80

**Table 3:** SAR imaging simulation parameters.

Parameter Name	Value	Parameter Name	Value
Radar center frequency	5.3 GHz	Antenna aperture	0.5 m
Transmitted pulse duration	1.5 μs	Radar flight altitude	1000 m
Effective radar velocity	60 m/s	Theoretical range resolution	0.25 m
Transmitted signal bandwidth	600 MHz	Theoretical azimuth resolution	0.25 m
Beam squint angle	0°	Average transmitted power	1 W
Radar incidence angle	30°	Radar gain	65 dB

## Data Availability

The data presented in this study are available in this article.
